# MAO-A Inhibition by Metaxalone Reverts IL-1β-Induced Inflammatory Phenotype in Microglial Cells

**DOI:** 10.3390/ijms22168425

**Published:** 2021-08-05

**Authors:** Giovanni Pallio, Angela D’Ascola, Luigi Cardia, Federica Mannino, Alessandra Bitto, Letteria Minutoli, Giacomo Picciolo, Violetta Squadrito, Natasha Irrera, Francesco Squadrito, Domenica Altavilla

**Affiliations:** 1 Department of Clinical and Experimental Medicine, University of Messina, Via C. Valeria, 98125 Messina, Italy; gpallio@unime.it (G.P.); adascola@unime.it (A.D.); luigi.cardia@unime.it (L.C.); fmannino@unime.it (F.M.); abitto@unime.it (A.B.); lminutoli@unime.it (L.M.); 2 SunNutraPharma, Academic Spin-Off Company of the University of Messina, Via C. Valeria, 98125 Messina, Italy; daltavilla@unime.it; 3 Department of Biomedical and Dental Sciences and Morphological and Functional Imaging, University of Messina, Via C. Valeria, 98125 Messina, Italy; giacomo.picciolo@unime.it; 4 Department of Human Pathology of Adult and Childhood “Gaetano Barresi”, University of Messina, Via C. Valeria, 98125 Messina, Italy; violettasquadrito@gmail.com

**Keywords:** neuroinflammation, microglia, metaxalone, MAO-A inhibition, antioxidant activity

## Abstract

Experimental and clinical studies have suggested that several neurological disorders are associated with the occurrence of central nervous system neuroinflammation. Metaxalone is an FDA-approved muscle relaxant that has been reported to inhibit monoamine oxidase A (MAO-A). The aim of this study was to investigate whether metaxalone might exert antioxidant and anti-inflammatory effects in HMC3 microglial cells. An inflammatory phenotype was induced in HMC3 microglial cells through stimulation with interleukin-1β (IL-1β). Control cells and IL-1β-stimulated cells were subsequently treated with metaxalone (10, 20, and 40 µM) for six hours. IL-1β stimulated the release of the pro-inflammatory cytokines tumor necrosis factor-alpha (TNF-α) and interleukin-6 (IL-6), but reduced the anti-inflammatory cytokine interleukin-13 (IL-13). The upstream signal consisted of an increased priming of nuclear factor-kB (NF-kB), blunted peroxisome proliferator-activated receptor gamma (PPARγ), and peroxisome proliferator-activated receptor gamma coactivator 1-alpha (PGC-1α) expression. IL-1β also augmented MAO-A expression/activity and malondialdehyde levels and decreased Nrf2 mRNA expression and protein levels. Metaxalone decreased MAO-A activity and expression, reduced NF-kB, TNF-α, and IL-6, enhanced IL-13, and also increased PPARγ, PGC-1α, and Nrf2 expression. The present experimental study suggests that metaxalone has potential for the treatment of several neurological disorders associated with neuroinflammation.

## 1. Introduction

Several neurological diseases are associated with neuroinflammation that represents a rational target of therapy and, recently, a marked microglia inflammation has been associated with fibromyalgia. Fibromyalgia (FM) is a poorly understood musculoskeletal disorder characterized by chronic widespread pain accompanied by fatigue, sleep/mood disturbances, and cognitive dysfunction [1]. After osteoarthritis, fibromyalgia is considered the second most common rheumatic disorder with a higher prevalence in women than in men [2]. Both its etiology and pathophysiology are still not fully known but central mechanisms are strongly involved, with an impairment of the central nervous system (CNS) in addition to the peripheral one [3,4]. The pain associated with fibromyalgia is related to the modulation and activation of different neural networks, including neurotransmitters, hormones, neuropeptides, cytokines, and chemokines that, working together, amplify pain perception [5]. This complex interactive mechanism is integrated in a tangle that involves the brain, spinal cord, and the peripheral tissues, which contributes to neurogenic inflammation. Patients affected by FM showed increased levels of neuropeptides and cytokines in their cerebrospinal fluid (CSF), such as substance P (SP) and interleukin-8 (IL-8) [6,7]; the release of neuropeptides and pro-inflammatory cytokines orchestrates neuroinflammation. The levels of IL-1, IL-6, IL-8, and TNF-α were found to also be increased in the blood of patients with fibromyalgia [8,9,10]: an association between IL-8, IL-6 and the severity of the disease has been demonstrated, thus showing that the release of pro-inflammatory cytokines negatively links to fibromyalgia and its prognosis [11]. Pregabalin, duloxetine, and milnacipran are approved in the US for the treatment of fibromyalgia and neuropathic pain, but their efficacy, safety, and compliance are not satisfactory [12,13]. One of the priorities of the treatment of FM is pain management. In this context, muscle relaxants are drugs whose mechanism of action is based on the reduction of muscle spasm, and although their use is not convincing, muscle relaxants are approved and well accepted in clinical practice as therapeutic adjuvants for the treatment of chronic musculoskeletal pain [14]. Metaxalone is a muscle relaxant, approved by the Food and Drug Administration as adjuvant therapy for the management of acute and painful musculoskeletal conditions, but it has no direct muscle relaxant effects [15]. The exact mechanism of action is not yet fully described, but it has been proposed as an inhibitor of monoamine oxidase (MAO) A [16]. Currently, the MAO–inflammation connection is not fully elucidated; nevertheless, in a previous study Deshwa et al. showed that co-exposure of cardiomyocytes to the pro-inflammatory cytokine IL-1β elicited MAO-related oxidative stress with subsequent mitochondrial dysfunction and endoplasmic reticulum stress [17]. Moreover, it has already been reported that lipopolysaccharide (LPS) stimulation resulted in the upregulation of both MAO isoforms via a signal transduction pathway that appears to involve NF-κB [18]. Furthermore, MAO upregulation triggered by LPS was observed in a rat periodontal disease model and treatment with a MAO inhibitor, phenelzine, was able to significantly reduce oxidative stress [19]. Another recent study showed that moclobemide, a reversible MAO-A inhibitor, was able to attenuate the inflammatory process in a rat model of ischemia-reperfusion injury [20]. Additionally, it has been reported that MAO-A is involved in ROS generation in alternatively activated monocytes/macrophages [21,22]. All these previous works demonstrated that MAO-A inhibition was able to decrease ROS generation and the ROS-activated inflammatory process in particular through NF-κB pathway inhibition. Several studies showed that inflammatory cytokines in fibromyalgia patients could drive disturbances in neural networks during the interaction of the nervous system with immune cells, which eventually could lead to an increase in central and peripheral sensitization as well as neuroinflammation [23]. For all these reasons, the aim of this study was to investigate the therapeutic potential of metaxalone in an in vitro experimental paradigm of microglial cell inflammation (obtained through IL-1β stimulation) [24] that mimics the phenomenon of neuroinflammation involved in several neurological disorders, including fibromyalgia.

## 2. Materials and Methods

### 2.1. Cell Treatment

Human microglial HMC3 cell line (CRL-3304) was obtained from ATCC (American Type Culture Collection; Manassas, VA, USA). Cells were cultured in Eagle’s minimum essential medium (EMEM) with a supplement of 10% fetal bovine serum (FBS) and 1% antibiotic mixture, and were incubated at 37 °C with 5% of CO_2_. Both medium and supplements were provided by ATCC (Manassas, VA, USA). Cells were seeded in 6-well plates at a density of 4 × 10^5^ cells/well. A stock solution of metaxalone (Sigma Aldrich, Milan, Italy) was prepared by dissolving metaxalone in DMSO. Sixteen hours after plating (time 0), metaxalone was added at the doses of 10, 20, and 40 μg/mL 1h following IL-1β stimulation (5 ng/mL; Sigma Aldrich, Milan, Italy). After 6 h cells were harvested for mRNA evaluation. In a separate set of plates, cells were incubated for 24 h before MTT assay to evaluate cell viability. The dose–response curve for IL-1β stimulation can be found at Supplemental Figure S1.

### 2.2. MTT Assay

MTT assay was used to evaluate cell viability. HMC3 cells were grown and incubated with IL-1β (5 ng/mL). Cells were treated with different concentrations of metaxalone (10, 20, and 40 μg/mL) in a 96-well plate at a density of 4 × 10^4^ cells/well for 24, 48, and 72 h to evaluate the cytotoxic effect of the drug; an additional group of cells was treated with staurosporine (100 nM; Sigma Aldrich, Milan, Italy) as a positive control. A mixture, constituted of 20 μL of the tetrazolium dye MTT 3-(4,5-dimethylthiazol-2-yl)-2,5-diphenyltetrazolium bromide (Sigma Aldrich, Milan, Italy), dissolved in sterile and filtered PBS, was added into each well 5 h before the end of the 24 h of incubation. Medium was then removed and the insoluble formazan crystals were dissolved through dimethyl sulfoxide (DMSO; 200 μL/well) addition, after 5 h. The difference in the values obtained at 540 and 620 nm of absorbance was used to evaluate the possible cytotoxic effect of metaxalone. The average of replicates was used for each group and the results were expressed as % of cell viability compared to untreated cells and reported as means and standard deviations [25,26,27].

### 2.3. Real-Time Quantitative PCR Amplification (RTqPCR)

Total RNA was extracted from human microglial HMC3 for RT-qPCR using Trizol LS reagent (Invitrogen, Carlsbad, CA, USA). Two µg of total RNA was reverse transcribed in a final volume of 20 μL using a Superscript VILO kit (Invitrogen, Carlsbad, CA, USA). The obtained cDNA (1 μL) was added to the EvaGreen qPCR Master Mix (Biotium Inc., Fremont, CA, USA), achieving a final volume of 20 μL per well. The final primer concentration used to carry out the analysis was 10 μM. Samples from each group were run in duplicate and β-actin was used as an endogenous control. Results were calculated using the 2^−ΔΔCT^ method and expressed as n-fold increase in gene expression using control group as calibrator [28,29]. Primers used for targets and reference genes are listed in Table 1:

### 2.4. Measurements of Cytokines by Enzyme-Linked Immunosorbent Assay (ELISA)

Tumor necrosis factor-alpha (TNF-α), interleukin-6 (IL-6) and interleukin-13 (IL-13) were measured in the cell culture supernatants. The products under investigation were measured using enzyme-linked immunosorbent assay (ELISA) kits (Abcam, Cambridge, UK), in agreement with the instructions reported by the manufacturer. All the samples were evaluated in duplicate and the obtained results were interpolated with the pertinent standard curves. To evaluate the sample, the means of the duplicated sample were used and expressed in pg/mL [30,31].

### 2.5. MAO-A Activity Assay

MAO-A activity was evaluated by a two-step bioluminescent assay methodology, as previously described [32], using a MAO-GLO^TM^ assay kit from Promega (Madison, WI, USA). The produced amount of signal (light intensity) was measured using a microplate luminometer (Multilabel Counter Victor3^TM^, PerkinElmer Life Sciences). Human recombinant MAO-A enzyme was used as a positive control. The results are given as relative light units after the background was subtracted.

### 2.6. Malondialdehyde Assay

Metaxalone antioxidant effects were examined in HMC3 cells by measuring malondialdehyde (MDA) levels as a marker of lipid peroxidation, as previously described in detail [33].

### 2.7. Western Blot Analysis

HMC3 cells were homogenized in RIPA buffer (25 mM Tris/HCl, pH 7.4; 1.0 mM EGTA; and 1.0 mM EDTA) with 1% of NP40, 0.5% of phenyl methylsulfonyl fluoride (PMSF), aprotinin, and leupeptin and pepstatin (10 μg/mL each) to perform protein extraction. Lysates were centrifuged at 15,000× *g* for 15 min at 4 °C and the supernatant was collected for protein determination using a specific kit (Bio-Rad DC; Bio-Rad, Richmond, CA, USA). Samples were denatured in a reducing buffer (62 mM Tris pH 6.8, 10% glycerol, 2% SDS, 5% β-mercaptoethanol, and 0.003% bromophenol blue) and proteins were separated by electrophoresis on SDS polyacrylamide gels. Following electrophoresis, samples were transferred onto a PVDF membrane (Amersham, Little Chalfont, UK) in a transfer buffer (39 mM glycine, 48 mM Tris pH 8.3, and 20% methanol) at 200 mA for 1 h. The obtained membranes were incubated with 5% non-fat dry milk in TBS/0.1% Tween for 1 h at room temperature, washed 3 times in TBS/0.1% Tween, and incubated with primary antibodies to detect pNF-κB, PPARγ, PGC-1α (Cell Signaling, Danvers, MA, USA), Nrf2, and MAO-A (Abcam, Cambridge, UK), diluted in TBS/0.1% Tween overnight at 4 °C. The day after, following 3 washes with TBS/0.1% Tween, membranes were incubated with secondary peroxidise-conjugated goat anti-mouse and anti-rabbit antibodies (KPL, Gaithersburg, MD, USA) for 1 h at room temperature. After washing, membranes were analyzed by the enhanced chemiluminescence system (LumiGlo reserve; Seracare, Milford, MA, USA). The protein signal was detected and quantified by scanning densitometry using a bio-image analysis system (C-DiGit, Li-cor, Lincoln, NE, USA). The results were expressed as relative integrated intensity and β-actin (Cell Signaling, Danvers, MA, USA) was used to confirm equal protein loading [34].

### 2.8. Statistical Analysis

Data are shown as mean ± SD and the reported values are the result of at least five experiments. To ensure reproducibility, all assays were replicated three times. The various groups were compared and evaluated using one-way ANOVA with Tukey post-test for comparison between the different groups. A *p* value < 0.05 was considered significant. Graphs were created and organized using GraphPad Prism (version 8.0 for macOS, San Diego, CA, USA).

## 3. Results

### 3.1. Metaxalone Reverts the Inflammatory Phenotype Induced by IL-1β in HMC3 Microglial Cells

**Figure 1:**
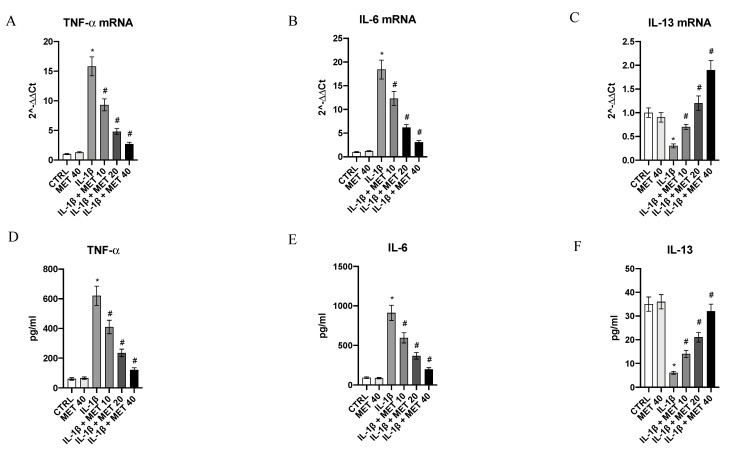
Effects of metaxalone on TNF-α (**A**), IL-6 (**B**), and IL-13 (**C**) mRNA expression. Effects of metaxalone on TNF-α (**D**), IL-6 (**E**), and IL-13 (**F**) protein levels. Values are expressed as the means ± SD. *, *p <* 0.0001 vs. CTRL; #, *p <* 0.0001 vs. IL-1β.

Inflammatory cytokines released by microglial cells orchestrate an inflammatory cascade that induces the development and progression of a long-term neuroinflammation, which plays a key role in the development and perpetuation of several neurological disorders, including fibromyalgia. For this reason, metaxalone’s effects on reducing an inflammatory phenotype were evaluated after stimulating HMC3 microglial cells with IL-1β. IL-1β challenge caused a significant increase in the expression of pro-inflammatory cytokines TNF-α and IL-6 (*p* < 0.0001 vs. CTRL) and a decrease in the anti-inflammatory cytokine IL-13 (*p <* 0.0001 vs. CTRL) expression, evaluated either as mRNA expression or protein levels (Figure 1). Metaxalone significantly reverted the inflammatory phenotype prompted by IL-1β in a dose-dependent manner (*p <* 0.0001 vs. IL-1β; Figure 1).

### 3.2. Effects of Metaxalone on Oxidative Stress

**Figure 2:**
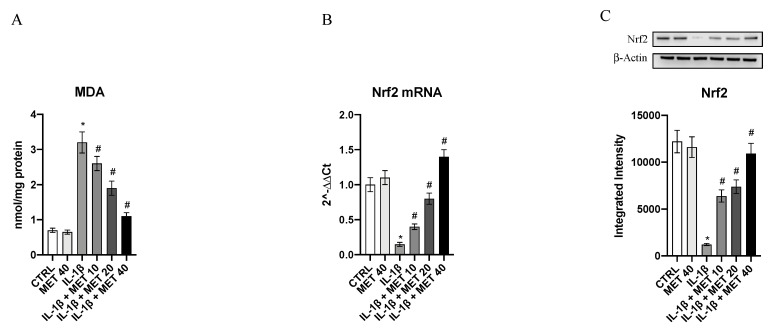
Effects of metaxalone on MDA generation (**A**), Nrf2 mRNA expression (**B**), and Nrf2 protein levels (**C**). Values are expressed as the means ± SD. *, *p <* 0.0001 vs. CTRL; #, *p <* 0.0001 vs. IL-1β.

A significant decrease in Nrf2 mRNA expression was observed in IL-1β-challenged cells compared to the control group, as a consequence of oxidative stress induction following IL-1β stimulation (*p* < 0.0001 vs. CTRL; Figure 2). Metaxalone treatment significantly upregulated Nrf2 gene expression in HMC3 cells (*p* < 0.0001 vs. IL-1β; Figure 2). Moreover, Nrf2 mature protein levels were significantly reduced in the IL-1β group compared to the control group (*p* < 0.0001 vs. CTRL; Figure 2). Furthermore, metaxalone treatment significantly increased Nrf2 levels in HMC3 cells after exposure to IL-1β stimulus in a dose-dependent manner (*p* < 0.0001 vs. IL-1β; Figure 2).

MDA levels were measured in the HMC3 cells to better characterize metaxalone antioxidant effects. Control cells showed low levels of MDA whereas IL-1β stimulation considerably increased MDA levels (*p* < 0.0001 vs. CTRL; Figure 2). Metaxalone treatment caused a significant reduction in MDA levels in HMC3 cells (*p* < 0.0001 vs. IL-1β; Figure 2), thus confirming its antioxidant properties.

### 3.3. Metaxalone Targets Upstream Signals That Trigger the Inflammatory Phenotype

**Figure 3:**
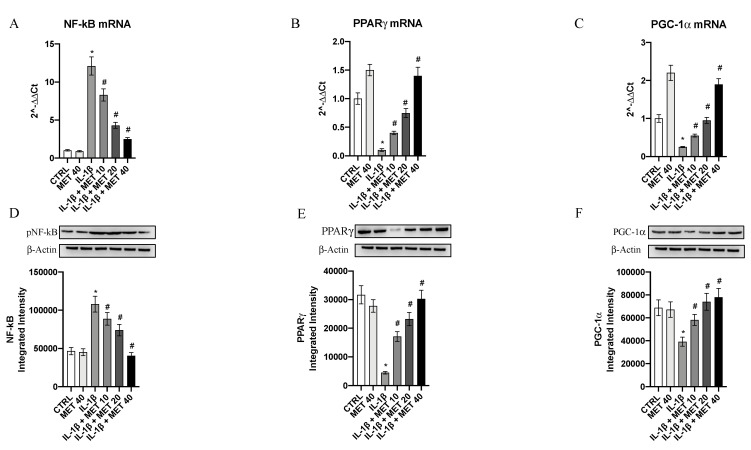
Effects of metaxalone on NF-kB (**A**), PPARγ (**B**), and PGC-1α (**C**) mRNA expression. Effects of metaxalone on pNF-kB (**D**), PPARγ (**E**), and PGC-1α (**F**) protein levels. Values are expressed as the means ± SD. *, *p <* 0.0001 vs. CTRL; #, *p <* 0.0001 vs. IL-1β.

The transcription factor NF-κB was markedly induced by IL-1β challenge in HMC3 cells (*p <* 0.0001 vs. CTRL; Figure 3); metaxalone significantly reduced its mRNA expression in a dose-dependent manner (*p <* 0.0001 vs. IL-1β; Figure 3). In addition, IL-1β stimulation also suppressed PPARγ and PGC-1α expression in microglial cells (*p <* 0.0001 vs. CTRL; Figure 3), and metaxalone was able to trigger a marked increase in the expression of both the nuclear receptor and its co-activator when compared to cell cultures challenged with IL-1β (*p <* 0.0001 vs. IL-1β; Figure 3).

The mature protein levels of inflammatory markers were measured in HMC3 cells stimulated with IL-1β to confirm metaxalone’s anti-inflammatory effects. *p*-NF-κB expression was markedly increased following IL-1β stimulus in HMC3 cells (*p <* 0.0001 vs. CTRL; Figure 3) and, by contrast, metaxalone treatment blunted the increase in *p*-NF-κB protein (*p <* 0.0001 vs. IL-1β; Figure 3). IL-1β challenge also significantly decreased PPARγ and PGC-1α protein expression in HMC3 cells compared to controls (*p <* 0.0001 vs. CTRL; Figure 3), whereas metaxalone treatment stimulated PPARγ and PGC-1α activation when compared to cell cultures challenged with IL-1β (*p <* 0.0001 vs. IL-1β; Figure 3), as demonstrated by their protein expression.

### 3.4. Metaxalone Reduces the Augmented MAO-A Expression and Activity Induced by IL-1β in Microglia Cells

**Figure 4:**
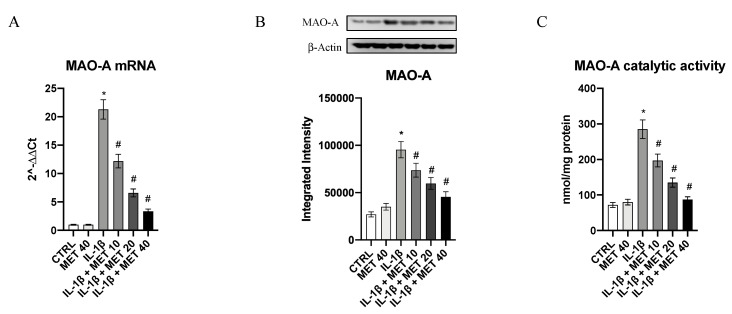
Effects of metaxalone on MAO-A mRNA expression (**A**), MAO-A protein levels (**B**), MAO-A activity (**C**). Values are expressed as the means ± SD. *, *p <* 0.0001 vs. CTRL; #, *p <* 0.0001 vs. IL1β.

MAO-A was constitutively expressed in microglia cells (Figure 4). IL-1β challenge caused a robust increase in MAO-A mRNA expression and protein levels (*p <* 0.0001 vs. CTRL; Figure 4A,B); metaxalone markedly reduced MAO-A mRNA expression in a dose-dependent fashion (*p <* 0.0001 vs. IL-1β; Figure 4A,B). MAO-A activity was also studied by using a chemiluminescence assay: IL-1β incubation resulted in an increased activity of MAO-A (*p <* 0.0001 vs. CTRL; Figure 4C). Thus, under our experimental condition, the triggering of an inflammatory phenotype in microglial cells was accompanied by an increase in MAO-A expression and, more interestingly, activity. As expected, metaxalone also suppressed MAO-A activity in a dose-dependent manner (*p <* 0.0001 vs. IL-1β; Figure 4C).

### 3.5. Effect of Metaxalone on HMC3 Microglial Cells Viability

**Figure 5:**
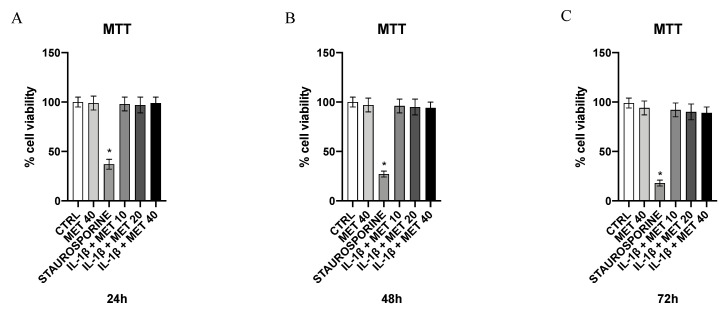
Effects of metaxalone on cell viability at 24 h (**A**), 48 h (**B**), and 72 h (**C**). Values are expressed as the means ± SD. *, *p <* 0.0001 vs. CTRL.

An MTT test was performed to evaluate the possible occurrence of a toxic effect following metaxalone treatment in microglial cells. Specifically, cells were incubated for 24, 48, and 72 h with the same concentrations of metaxalone, 10, 20, and 40 μg/mL. As shown in Figure 5, metaxalone did not produce any toxic effect in the treated cells and related viability percentage kept similar to control at all reported concentrations and time points, whereas the staurosporine-treated group (positive control) showed a significant reduction in cell viability at 24, 48, and 72 h (Figure 5).

## 4. Discussion

Several therapeutic approaches for the treatment of neurological diseases characterized by neuroinflammation, like fibromyalgia, have been proposed [35]. However, the rate of therapeutic failure with the available drugs is still very high, and this justifies the exploration of innovative treatments eventually emerging from robust preclinical data on the underlying physiopathology [36].

Recently, a new scenario has caught researchers’ interest: neuroinflammation in the CNS may play an important role in amplifying pain perception and the correlated depression symptoms [37]. An increase in pro-inflammatory chemokine and cytokine levels would cause a sensitization of central nociceptors, causing negative effects on symptoms and worsening the prognosis of the affected patients. In fact, patients with fibromyalgia showed an increase in interleukin and TNF-α levels in serum and plasma, which contribute not only to the exacerbation of inflammatory response but also to pain.

Microglial cells are resident macrophages responsible for the mechanisms of defense and repair of nerve tissue [38]. However, these cells, once activated, can contribute to neuroinflammation and especially to the development of numerous neurodegenerative diseases. In fact, microglial cells release and activate several molecules involved in inflammatory processes such as iNOS, IL-1β, TNF-α, COX-1, COX-2, reactive oxygen species (ROS), and potentially neurotoxic compounds that cause neuronal dysfunctions and cell death [39]. Therefore, despite the activation of microglia and neuroinflammation they might have a neuroprotective role; on the other hand, an exaggerated activation, resulting in a storm of pro-inflammatory cytokines, can contribute to the onset of neurological symptoms and neurodegenerative processes [40,41]. This also holds true for fibromyalgia; therefore, a possible therapeutic approach could aim at inhibiting the pro-inflammatory mediators produced by microglia.

Metaxalone (5-((3,5-dimethylphenoxy) methyl)-2-oxazolidinone) is a muscle relaxant indicated for the treatment of acute musculoskeletal pain, and even if its mechanism of action is still not fully known, a general sedation of the nervous system seems to be involved [42]. Metaxalone has been approved by the Food and Drug Administration (FDA) as an adjuvant therapy in the treatment of acute and painful musculoskeletal conditions, and is commonly prescribed as a muscle relaxant, although it has no direct muscle relaxant effects. However, it has been proposed that it may inhibit monoamine oxidase (MAO) A; this enzyme, besides being involved in the metabolism of serotonin, has been linked to several pathological conditions characterized by exaggerated oxidative stress and inflammation [43,44]; therefore, its inhibition may represent a strategy to design anti-inflammatory drugs.

A recent report has unmasked the ability of metaxalone to exert anti-inflammatory activity; in fact, the drug caused a robust reduction in several inflammatory cytokines in LPS-stimulated mouse macrophages [45]. However, this preliminary report used a cell line that has a scarce translational potential and did not address any attempt to dissect out the underlying mode of action.

In the present study, metaxalone’s anti-inflammatory effect was confirmed in microglial cells, the true resident macrophages of the CNS. Metaxalone suppressed the inflammatory phenotype triggered by IL-1β in a dose-dependent manner. Interestingly, we investigated the upstream signals that might be interrupted by metaxalone. The drug inhibited the augmented expression of NF-kB and increased the reduced expression of PPARγ in microglia cells. Indeed, activation of this nuclear receptor has been linked to a strong downregulation of the inflammatory phenotype in microglial cells [46,47,48]. Therefore, our hypothesis is that metaxalone finely tunes in an anti-inflammatory modality the upstream mechanisms that are involved in the inflammatory cascade.

This mechanism is likely due to the ability of metaxalone to inhibit the augmented expression and activity of MAO-A. Indeed, blockade of this enzyme has been shown to cause marked antioxidant and anti-inflammatory activity [49,50]. Accordingly, our results have shown that IL-1β stimulation causes an increase in oxidative stress in HMC3 cells. Metaxalone treatment significantly reduced oxidative stress markers (MDA) and upregulated the mRNA expression and protein levels of Nrf2, chief regulator of the antioxidant system, when compared to cell cultures challenged with IL-1β alone.

In conclusion, this study demonstrates for the first time that metaxalone has important antioxidant and anti-inflammatory effects; in addition, metaxalone is currently on the market and severe side effects have not been reported. Therefore, it could be used for the management of neuroinflammation and pain, even in patients affected by fibromyalgia. However, further studies will be needed to confirm these hypotheses and to define the complex mechanism of action of metaxalone for its possible use in clinical practice.

## Figures and Tables

**Table 1:** Primer list.

Gene	Sequence
*β-actin*	*Fw:5′AGAGCTACGAGCTGCCTGAC3′*
	*Rw:5′AGCACTGTGTTGGCGTACAG3′*
*IL-6*	*Fw:5′TTCGGTCCAGTTGCCTTCTC3′*
	*Rw:5′CAGCTCTGGCTTGTTCCTCA3′*
*IL-13*	*Fw:5′CATGGCGCTTTTGTTGACCA 3′*
	*Rw:5′AGCTGTCAGGTTGATGCTCC3′*
*NF-kB*	*Fw:5′CCTGGATGACTCTTGGGAAA3′*
	*Rw:5′TCAGCCAGCTGTTTCATGTC3′*
*PPAR-γ*	*Fw:5′TCGACCAGCTGAATCCAGAG3′*
	*Rw:5′GGGGGTGATGTGTTTGAACTTG3′*
*PGC-1a*	*Fw:5′CATGTGCAACCAGGACTCTGA3′*
	*Rw:5 GCGCATCAAATGAGGGCAAT3′*
*TNF-α*	*Fw:5′CAGAGGGCCTGTACCTCATC3′*
	*Rw:5′GGAAGACCCCTCCCAGATAG3′*
*MAO-A*	*Fw:5′ATGACACCAAGCCAGATGGG3′*
	*Rw:5′TCAGCAGGCCAGAAACAGAG3′*
*Nrf2*	*Fw:5′CTCCACAGAAGACCCCAACC3′*
	*Rw:5′TCTGCAATTCTGAGCAGCCA3′*

## Supplementary Materials

The following are available online at https://www.mdpi.com/article/10.3390/ijms22168425/s1.
