# Supplementary figures and images for: MAO-A Inhibition by Metaxalone Reverts IL-1β-Induced Inflammatory Phenotype in Microglial Cells
Source: Int J Mol Sci. 2021 Aug 5;22(16):8425. doi: 10.3390/ijms22168425 (PMC8395141; doi:10.3390/ijms22168425)

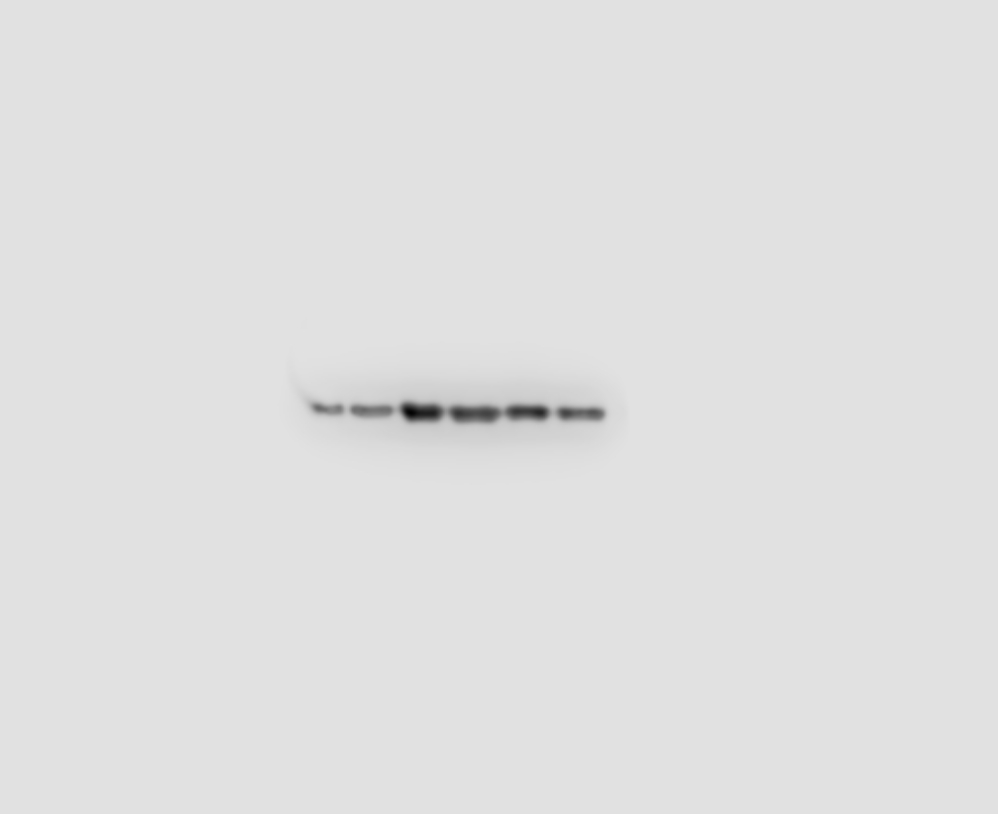

Supplement: Supplementary file 1 [file ijms-22-08425-s001.zip › MAO-A.jpg]

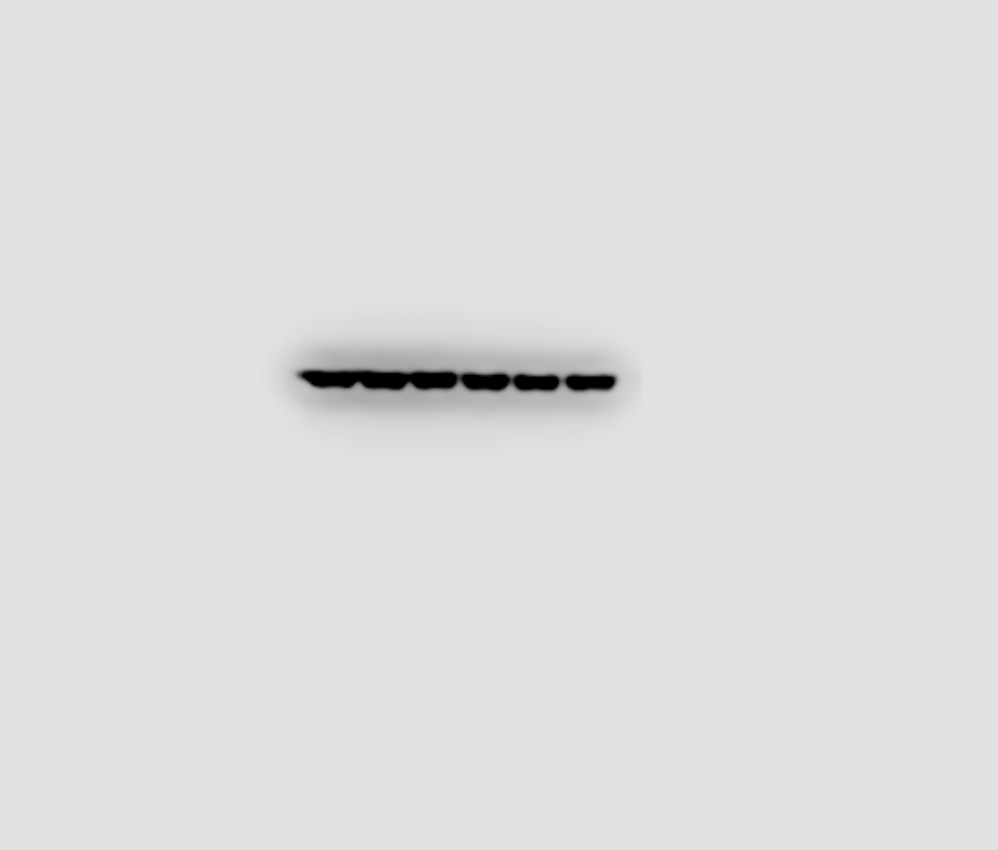

Supplement: Supplementary file 1 [file ijms-22-08425-s001.zip › Actin.jpg]
